# RNF20 affects porcine adipocyte differentiation via regulation of mitotic clonal expansion

**DOI:** 10.1111/cpr.13131

**Published:** 2021-10-14

**Authors:** Ying Zhao, Jianfei Pan, Chunwei Cao, Xiaojuan Liang, Shulin Yang, Lulu Liu, Cong Tao, Jianguo Zhao, Yanfang Wang

**Affiliations:** ^1^ State Key Laboratory of Animal Nutrition Institute of Animal Science Chinese Academy of Agricultural Sciences Beijing China; ^2^ Guangdong Provincial Key Laboratory of Malignant Tumor Epigenetics and Gene Regulation Guangdong‐Hong Kong Joint Laboratory for RNA Medicine Sun Yat‐Sen Memorial Hospital Sun Yat‐Sen University Guangzhou China; ^3^ State Key Laboratory of Stem Cell and Reproductive Biology Institute of Zoology Chinese Academy of Sciences Beijing China; ^4^ Savaid Medical School University of Chinese Academy of Sciences Beijing China

**Keywords:** RNF20, C/EBPβ, mitotic clonal expansion, porcine preadipocyte differentiation

## Abstract

**Objectives:**

RNF20 is recognized as a main E3 ligase for monoubiquitination of histone H2B at lysine 120 (H2Bub). The critical role of RNF20 and H2Bub in various molecular events, such as DNA replication, RNA transcription, and DNA damage response, has been widely investigated and documented. However, its role in porcine adipogenesis remains unknown. In this study, we aimed to clarify the effect of RNF20 on porcine preadipocyte differentiation.

**Materials and Methods:**

Backfat tissues from fat‐type pigs (Bama and Meishan) and lean‐type pigs (Yorkshire and Landrace) were collected to detect the expression level of RNF20. Preadipocytes were isolated from Bama piglets and induced to differentiation. Small interfering RNAs were applied to deplete RNF20. Oil Red O staining, quantitative real‐time PCR, RNA‐seq, Western blot analysis, and EdU assays were performed to study the regulatory mechanism of RNF20 during adipogenesis.

**Results:**

We found that the expression levels of RNF20 and H2Bub were significantly higher in backfat tissues from fat‐type pigs than in those from lean‐type pigs. Consistently, the significantly induced expression of RNF20 and H2Bub was also observed in porcine differentiated adipocytes. In addition, knockdown of RNF20 greatly inhibited porcine adipogenesis, as evidenced by dramatically decreased lipid droplet formation and lower expression levels of adipogenic transcription masters in *RNF20* knockdown cells. Mechanistically, the depletion of RNF20 decreases the cell proliferation and the level of p‐C/EBPβ via the Ras‐Raf‐MEK1/2‐ERK1/2 cascade pathway at the mitotic clonal expansion phase and therefore suppresses cell differentiation.

**Conclusions:**

Our results demonstrate that RNF20 is required for porcine preadipocyte differentiation.

## Graphical abstract

RNF20 is required for porcine preadipocyte differentiation. Our data indicate that the depletion of RNF20 decreases the cell proliferation and the level of p‐C/EBPβ via the Ras‐Raf‐MEK1/2‐ERK1/2 cascade pathway at the mitotic clonal expansion phase and therefore suppresses cell differentiation.

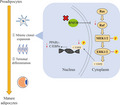

## 1 INTRODUCTION

Pigs are the most important meat livestock worldwide. Fat deposition‐related traits, including backfat thickness, carcass leanness, feed efficiency, and meat quality, are of economic importance in swine breeding. Understanding the genetic basis of fat deposition is necessary for a successful breeding program; therefore, the identification of fat trait‐related genes and networks has become a hotspot in the research field of pig genetics and breeding. Over the past several decades, especially with the recent development of more advanced methodologies, many fat deposition‐related genes have been identified in pigs, and they can be found in many review articles.^1^ However, most of these candidate genes were screened by quantitative trait locus (QTL) mapping, genome‐wide association studies (GWASs), single nucleotide polymorphism (SNP) association analyses, and transcription‐based studies, and the detailed effects of these candidates on adipogenesis and lipogenesis in pigs remain unclear.

Adipogenesis, which is separate from lipogenesis, is a complex process of cell differentiation during that fibroblast‐like preadipocytes develop into mature adipocytes. It is crucial for the development of adipose tissue. Adipogenesis includes two phases: the phase of mitotic clonal expansion (MCE) and the phase of differentiation.^2^, ^3^ The MCE phase has been reported to be a necessary step for terminal differentiation,^4^ during that growth‐arrested preadipocytes synchronously reenter the cell cycle and undergo approximately two rounds of division.^5^, ^6^, ^7^ As a member of the C/EBP family of transcription factors, C/EBPβ was observed to be expressed immediately at the early MCE stage (usually 2–4 h after induction) but without DNA‐binding activity at this time. DNA‐binding activity acquisition of C/EBPβ occurred at approximately 14 h and peaked at approximately 24 h after induction.^8^ Having acquired DNA‐binding properties, C/EBPβ binds to the C/EBP regulatory elements of peroxisome proliferator‐activated receptor‐γ (PPARγ) and C/EBPα and activates their transcriptional activities, which seem to function as terminators of MCE.^4^, ^8^ Although these master transcriptional regulators have been proven to exert central roles in adipogenesis, identification of the involved genes and networks, as well as exploration of the genetic regulatory mechanisms that control porcine adipogenesis, is required to better understand fat deposition in pigs.

Ring finger protein 20 (RNF20) has been recognized as a main E3 ligase for the monoubiquitination of histone H2B lysine 120 (hereafter referred to as H2Bub).^9^, ^10^ The critical role of RNF20 and its mediation of H2Bub in various molecular events have been widely investigated, such as DNA replication,^11^ DNA damage response and repair,^12^, ^13^ transcription,^14^, ^15^ and RNA processing.^16^, ^17^ RNF20 and H2Bub have been shown to be required for controlling the differentiation of various cells, including osteogenesis,^18^ myogenesis,^19^ and astrocytic differentiation.^20^ The function of RNF20 in lipid metabolism has also been documented.^21^, ^22^, ^23^, ^24^ A study with the murine 3T3‐L1 cell line, a well‐established in vitro model of adipogenesis, revealed that RNF20 plays a role in differentiation by stimulating ubiquitin‐proteasome‐dependent degradation of AP‐2α.^21^ RNF20 has been reported to act as a negative regulator of hepatic fatty acid metabolism through degradation of a transcription factor, sterol regulatory element‐binding protein 1c (SREBP1c).^22^ Recent studies with 3T3‐L1 cells and *Rnf20* ^+/−^ mice revealed that RNF20 promotes adipogenesis by potentiating the transcriptional activity of PPARγ via promoting nuclear corepressor 1 (NCoR1) degradation.^23^ Our previous study showed that *Rnf20* ablation impaired adipose tissue development in mice and that an agonist of PPARγ, rosiglitazone, could partially rescue the phenotype.^24^ All of these studies are based on mice, and little is known about the function of RNF20 in regulating porcine adipocyte differentiation. In our previous work, we isolated the porcine *RNF20* gene and found that RNF20 is highly expressed in adipose tissues in pigs,^25^ suggesting its role in pig adipogenesis.

The aim of the present study was to examine the expression level of RNF20 in backfat tissues from fat‐type and lean‐type pigs and investigate the role of the *RNF20* gene in porcine adipogenesis, especially in the MCE phase of adipocyte differentiation.

## 2 MATERIALS AND METHODS

### 2.1 Adipose tissues

Backfat tissues of Bama, Meishan, Yorkshire, and Landrace pigs at 6 months of age were collected and kept in our lab. All samples were frozen in liquid nitrogen immediately and stored at −80°C until further use.

### 2.2 Preadipocytes isolation, culture, and in‐vitro differentiation

Porcine preadipocytes were harvested from 7‐day‐old Bama piglets of subcutaneous adipose tissues, minced, and digested with 2 mg/mL collagenase type I (Sigma‐Aldrich, St. Louis, MO, USA) in D‐Hanks (Solarbio, Beijing China). Cells were cultured in Dulbecco's modified DMEM/F12 (HyClone, South Logan, UT, USA) plus 10% fetal bovine serum (FBS, HyClone, South Logan, UT, USA) and 1% penicillin‐streptomycin (PS, Sigma Aldrich, St. Louis, MO, USA) for 24 h at 38.5°C. The complete medium (DMEM/F12 plus 10% FBS and 1% PS) was changed every two or three days. After reaching 90% confluence, the cells were transferred to six‐well plates (Corning, NY, USA) at a density of 6 × 10^5^ cells in 2 mL per well. Cells were grown to confluence, and the standard culture medium was removed and replaced with induction medium for 5 days. On day 5, half of the induction medium was removed, and the same volume of mature medium was added. On day 6, the cells were switched to mature medium and cultured for 2 days. On day 8, fully differentiated adipocytes were used for the following experiments. The induction medium was DMEM (HyClone, South Logan, UT, USA) containing 10% FBS, 20 mM HEPES pH 7.4 (Solarbio, Beijing, China), 5 μg/mL insulin (Macgene, Beijing, China), 17 μM pantothenate (Sigma‐Aldrich, St. Louis, MO, USA), 33 μM biotin (Sigma‐Aldrich, St. Louis, MO, USA), 0.25 mM 3‐isobutyl‐1‐methylxanthine (IBMX; Sigma‐Aldrich, St. Louis, MO, USA), 1 μM dexamethasone (DEX, Sigma‐Aldrich, St. Louis, MO, USA), and 1 μM rosiglitazone (Sigma‐Aldrich, St. Louis, MO, USA). The mature medium was DMEM containing 10% FBS, 20 mM HEPES, 17 μM pantothenate, 33 μM biotin, 5 μg/mL insulin, and 1 μM DEX.

### 2.3 Oil Red O staining

Mature adipocytes were stained with Oil Red O (ORO) to confirm differentiation efficiency. Cells were fixed with 4% paraformaldehyde (PFA) (Solarbio, Beijing, China) for 30 min at room temperature or overnight at 4°C. Next, the working solution of ORO (Sigma‐Aldrich, St. Louis, MO, USA) (Oil Red O: deionized water = 4:6) was prepared and kept away from light at room temperature. Then, 60% isopropanol was used to wash the cells. The cells were stained with the working solution for 15–20 min at room temperature. After staining, the cells were washed twice with distilled water. Finally, the cells were examined microscopically and photographed (Nikon, Tokyo, Japan).

### 2.4 RNA isolation and reverse transcription (RT)

Total RNA was extracted from cells using RNAiso (Takara, Tokyo, Japan) according to the manufacturer's instructions. The purity and concentration of total RNA were measured by a spectrophotometer (Nanodrop 2000, Thermo Fisher Scientific, Waltham, MA, USA) at 260 and 280 nm. Absorption ratios (260/280 nm) of all samples were between 1.80 and 2.00. cDNA was synthesized using a Primer Script^TM^ RT Reagent Kit with gDNA Eraser (Takara, Tokyo, Japan).

### 2.5 Quantitative real‐time PCR analysis (qPCR)

Transcript levels were determined by qPCR using Quant Studio 3 (Thermo Fisher Scientific, Waltham, MA, USA). Gene‐specific primers were designed using the online website Primer 3 (v. 0. 4. 0) (http://bioinfo.ut.ee/primer3‐0.4.0/). Primer sequences are shown in Table S1. PCR protocol: 95°C for 5 min, followed by 40 cycles of 95°C for 5 s and 60°C for 34 s. Relative gene expression was calculated by using the 2^−ΔΔCt^ (the difference between the threshold cycles) method. *18S* was used as a housekeeping gene. All measurements were repeated ≥3 times.

### 2.6 Western blot analysis

Total proteins were isolated from cells using M‐PER™ Mammalian Protein Extraction Reagent (Thermo Fisher Scientific, Waltham, MA, USA) supplemented with protease and phosphatase inhibitors (Roche, Basel, Switzerland). Tissue proteins were extracted by using T‐PER™ Tissue Protein Extraction Reagent (Thermo Fisher Scientific, Waltham, MA, USA) supplemented with protease and phosphatase inhibitors. Proteins were boiled at 100°C for 10 min and then resolved by electrophoresis through a 10% SDS‐PAGE gel (EpiZyme, Beijing, China). Separated proteins were transferred onto nitrocellulose membranes (Merck Millipore, Billerica, MA, USA), blocked with blocking buffer (5% fat‐free milk) for 2 h at temperature and incubated overnight at 4°C with the following primary antibodies: RNF20 (Proteintech, Wuhan, China). H2Bub, PPARγ, CEBPα, p‐C/EBPβ (Thr235), PCNA, MEK1/2, p‐MEK1/2 (Ser217/221), ERK1/2, p‐ERK1/2 (Thr202/204), β‐Tubulin, and H2B (CST, Danvers, MA, USA), anti‐histone H3 (trimethyl K4), anti‐histone H3 (trimethyl K79) (Abcam, Melbourne, VIC, Australia), and C/EBPβ (Santa Cruz Biotechnology, Dallas, TX, USA). The blots were developed using goat anti‐rabbit IgG HRP‐linked secondary antibody or goat anti‐mouse IgG HRP‐linked secondary antibody (CST, Danvers, MA, USA) and detected using a FluorChem M Fluorescent Imaging System (Tanon 5200, Tanon Science & Technology Co., Ltd., Shanghai, China) with Tanon™ High‐sig ECL Western Blotting Substrate (Tanon Science & Technology Co., Ltd., Shanghai, China). Blot signaling was quantified by standard densitometric analysis using ImageJ v. 47 (National Institute of Health, Bethesda, MD, USA).

### 2.7 Knockdown of RNF20 by siRNA transfection

In the knockdown experiments, gene‐specific small interfering RNA (siRNA) was designed by using the complete cDNA sequence of the porcine *RNF20* gene (NCBI Gene ID: 100154259), appropriate small interfering RNA (siRNA) target sites were selected, and the corresponding primers were designed and synthesized. A nonsense codon sequence was used as a negative control. The specific siRNAs for the porcine *RNF20* gene (siRNF20) and negative control (siNC) used in this experiment were purchased from Gene Pharma (Shanghai, China). Sequence information for the specific siRNAs is provided in Table S2. Porcine preadipocytes were transfected with siRNA using a Lipofectamine RNAiMAX reagent kit (Invitrogen, Carlsbad, CA, USA). The efficiency of transfection was assessed at 48 h and 72 h by Western blot.

### 2.8 RNA‐seq and differentially expressed gene (DEG) screening

Sequencing library preparation and RNA‐seq were conducted at Shanghai Personal Biotechnology Co., Ltd. RNA samples with high purity (OD260/280 ≥ 2.0) and high integrity (RIN > 8) were used for cDNA library construction. More detailed information on cDNA library construction, sequencing of PE libraries, quality control, read mapping, and FPKM calculations can be found in a previous study.^26^ DEGs were screened by the criteria of a *p*‐value <0.05 and a fold change (FC) >2.0.

### 2.9 Kyoto encyclopedia of genes and genomes (KEGG) analysis and signaling network analyses

KEGG enrichment analysis of upregulated and downregulated genes was performed using the “DAVID 6.8” Functional Annotation Tool (https://david.ncifcrf.gov/). Pathways with *p*‐values <0.05 were regarded as statistically significant. Signaling networks of DEGs were analyzed using the online tool Metascape (https://metascape.org/gp/index.html#/main/step1).

### 2.10 Cell proliferation analysis

5′‐ethynyl‐2′‐deoxyuridine (EdU) incorporation was assessed using the Cell‐Light EdU Apollo567 In Vitro Kit (Guangzhou RiboBio, Guangzhou, China) according to the manufacturer's recommendation. Briefly, porcine preadipocytes were allowed to reach confluency (referred to as day −2) in a 48‐well cell imaging plate (Corning, NY, USA) and maintained at confluency for 2 days (referred to as day 0). Differentiation was initiated at day 0, and EdU was added at a final concentration of 5 μM after 16 h of differentiation. After a total of 40 h of differentiation, cells were fixed in 4% PAF for 30 min at room temperature and permeabilized with 0.5% Triton X‐100. Subsequently, 1× Apollo reaction cocktail was added to the cells and incubated for 30 min, and then the cells were stained with Hoechst 33342 for DNA content analysis. Finally, EdU‐positive cells (EdU^+^) were visualized under a fluorescence microscope (Nikon, Tokyo, Japan). The analysis of porcine preadipocyte proliferation (ratio of EdU^+^ cells to all cells) was performed using images of randomly selected fields (*n* = 16) obtained under a fluorescence microscope.

### 2.11 Statistical analysis

GraphPad Prism (Version 8, La Jolla, CA, USA) was used for statistical analysis. Statistical comparisons between two groups were made using the unpaired 2‐tailed Student's *t* test. In all cases, levels of statistical significance were set at *p < 0.05, **p < 0.01, and ***p < 0.001. All data are presented as the mean ± standard error mean (SEM). Each mean value was obtained from at least three independent experiments.

## 3 RESULTS

### 3.1 RNF20 expression is significantly higher in backfat from fat‐type pigs

**FIGURE 1:**
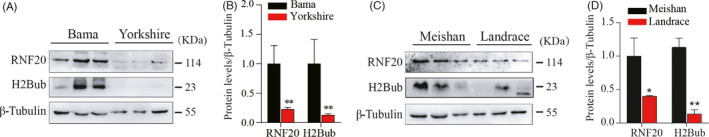
RNF20 was highly expressed in backfat tissues of fat‐type pigs. (A) Western blot was used to measure the levels of RNF20 and H2Bub in backfat tissues from 6‐month‐old Bama and Yorkshire pigs. (B) Quantitative data from (A). (C) Western blot analysis of the levels of RNF20 and H2Bub in backfat tissues from 6‐month‐old Meishan and Landrace pigs. (D) Quantitative data from (C). *n* = 3. Results are presented as mean ± SEM. *p < 0.05, **p < 0.01, for differences between pig breeds

It has been reported that the expression levels of RNF20 in white adipose tissues (WATs) from high‐fat diet‐fed mice are significantly higher than those from chow diet‐fed mice.^24^ It is of interest to test the expression levels of RNF20 in adipose tissues from fat‐type and lean‐type pigs. We examined the expression levels of RNF20 in backfat tissues from Bama pigs, a fat‐type Chinese native pig breed, and Yorkshire, a well‐known commercial lean‐type pig. We found that the expression of RNF20, as well as the levels of H2Bub, were dramatically higher in backfat tissues from Bama pigs than in those from Yorkshire pigs (Figure 1A). The quantitative Western blot data showed that the difference reached a significance level (Figure 1B). Consistently, significantly higher expression levels of RNF20 and H2Bub were also observed in other fat‐type pig breeds, Meishan pigs. (Figure 1C,D). These data suggest a potential positive role of RNF20 in the formation of adipose tissue.

### 3.2 RNF20 is dramatically induced in differentiated adipocytes

**FIGURE 2:**
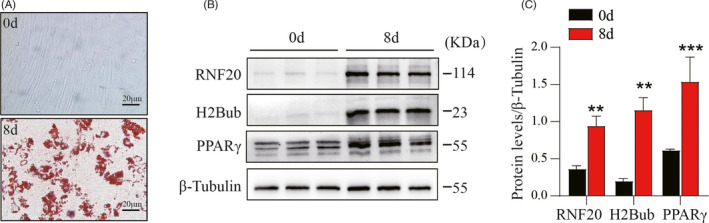
RNF20 was significantly induced after porcine preadipocyte differentiation. (A) Representative images of ORO staining of lipid droplets in porcine undifferentiated preadipocytes (0 day) and differentiated adipocytes (8 days of differentiation). Scare bar: 20 μm. (B) Western blotting was used to detect the levels of RNF20, H2Bub and adipogenic marker (PPARγ). Note that these proteins were greatly increased after induction for 8 days. (C) Quantitative analysis for (B). The expression levels of these proteins were normalized to β‐Tubulin. *n* = 3. Data are shown as the mean ± SEM, **p < 0.01 and ***p < 0.001

To examine the effect of RNF20 on adipocyte differentiation in pigs, primary preadipocytes were isolated from 7‐day‐old Bama piglets and differentiated into mature white adipocytes. Successful differentiation was proved by positive ORO staining (Figure 2A) and a dramatic increase in the adipogenic transcription factor PPARγ (Figure 2B,C) after 8 days of differentiation. Western blot analysis showed that the expression levels of RNF20 and H2Bub were remarkably induced after differentiation (Figure 2B,C). The significant induction of RNF20 and H2Bub in mature white adipocytes suggested that RNF20 possibly plays a positive role in porcine adipogenesis.

### 3.3 RNF20 is required for porcine preadipocyte differentiation

**FIGURE 3:**
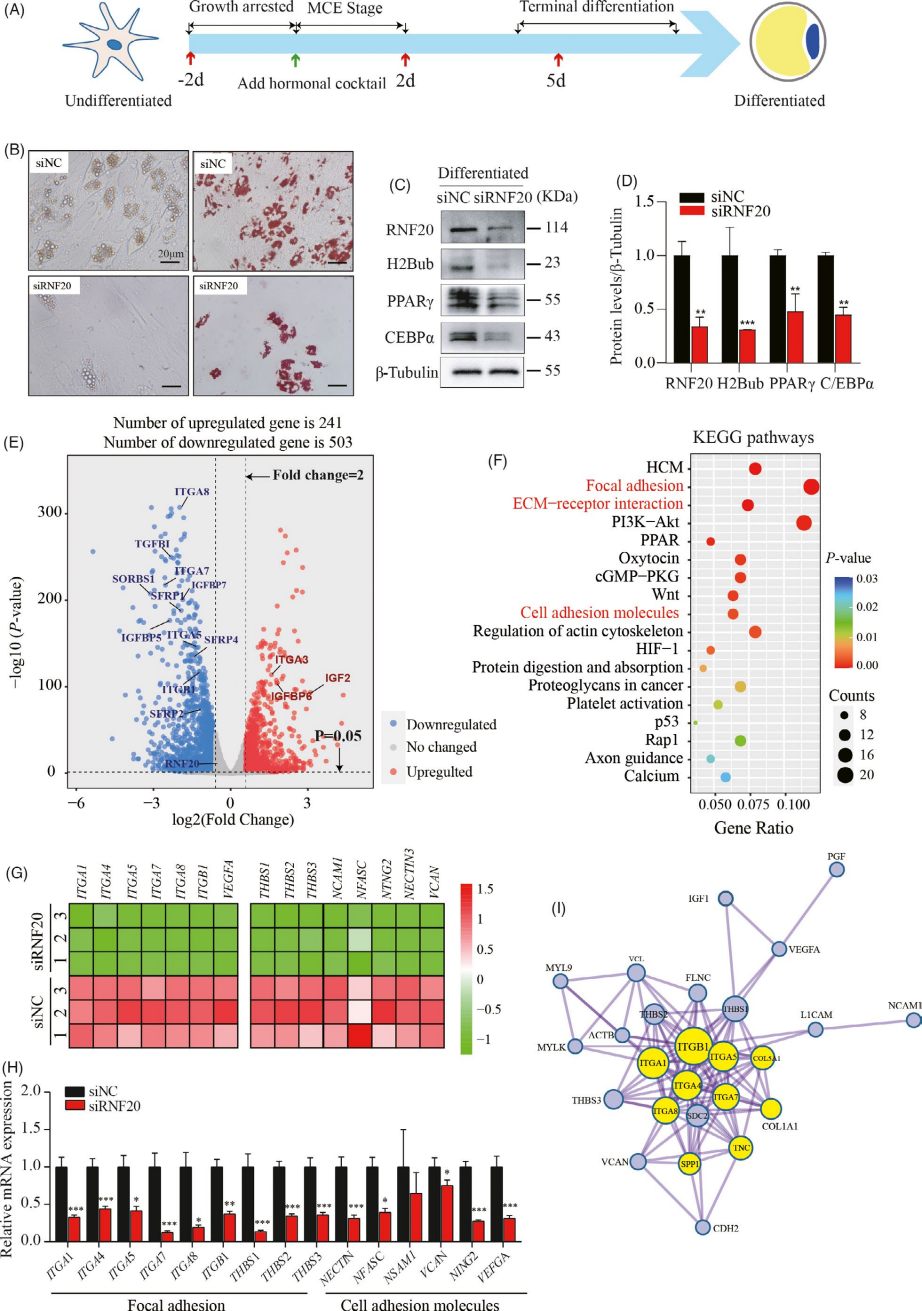
Knockdown of RNF20 expression suppressed porcine preadipocyte differentiation. (A) Schematic diagrams of experimental programs for exploring the effect of RNF20 on porcine preadipocyte differentiation. The green arrow indicates that growth‐arrested cells were induced with a hormonal cocktail at day 0. Red arrows indicate that cells were transfected with siNC or siRNF20 at days −2, 2, and 5 during differentiation. (B) The efficiency of adipocyte differentiation was dramatically decreased in siRNF20 cells based on lipid accumulation under bright‐field microscopy (left panel) and ORO staining (right panel). Scale bar: 20 μm. (C) The expression levels of the indicated proteins (RNF20 and H2Bub) and adipogenic markers (C/EBPα and PPARγ) were measured in siNC‐ and siRNF20‐transfected differentiated adipocytes (after 8 days of induction). β‐Tubulin was the loading control. (D) Data quantification of panel (C). Western blots representative of three independent experiments. *p < 0.05, **p < 0.01 vs. vehicle treatment. (E) Both siNC and siRNF20 differentiated adipocytes were collected, and RNA was purified for RNA‐seq. *n* = 3. Volcano plot of the genes displaying significantly different expression between two cells. Blue dots represent the significantly downregulated genes (503 genes, *p* < 0.05, FC >2.0), while red dots represent the significantly upregulated genes (241 genes, *p* < 0.05, FC <−2) in siRNF20 cells. (F) Bubble diagram showing the top enriched functional pathways of downregulated genes in siRNF20 cells, which were analyzed by KEGG pathway analysis. Y‐axis label represents pathway and X‐axis label represents gene ratio (gene ratio = amount of the genes enriched in the pathway/amount of all genes in background gene set). The size and color of the bubble represent the number of genes enriched in the pathway and enrichment significance, respectively. (G) Heatmap of the downregulated genes in focal adhesion and cell adhesion molecule pathways. (H) qPCR validation of the selected DEGs (G) in siNC‐ and siRNF20‐transfected differentiated adipocytes. *18S* was used as an internal control. *n* = 3. Results are shown as the mean ± SEM, *p < 0.05, **p < 0.01. (I) Twenty‐six genes involved in focal adhesion and cell adhesion molecule signaling pathways were used to build the molecular network. Note: The light purple circles represent the genes related to focal adhesion, while the yellow circles represent the genes related to cell adhesion molecules

To further investigate whether the lack of RNF20 affects porcine preadipocyte differentiation, three small interfering RNAs (siRNAs) specific for the porcine *RNF20* gene were designed and used to transfect porcine primary preadipocytes. The knockdown efficiency of three siRNAs was detected by Western Blot analysis at 48 h and 72 h post‐transfection in preadipocytes. Our data showed that siRNF20‐2 dramatically decreased the expression level of RNF20 at 72 h after transfection (Figure S1A,B), and this siRNA was referred to as siRNF20 and used for the following studies. Porcine preadipocytes were transfected with siRNF20 or negative control, named siNC, three times at different time points (day −2, day 2, and day 5) during adipogenesis, and a schematic overview of this experiment can be found in Figure 3A. Our data showed that strikingly fewer lipid droplets were formed in siRNF20‐transfected cells than in siNC‐transfected cells by morphology observation under brightfield microscopy (Figure 3B, left column), and the adipogenesis defect of siRNF20‐transfected cells was further confirmed by ORO staining (Figure 3B, right column). Moreover, the protein levels of the transcriptional markers, PPARγ and CEBPα, were significantly reduced in siRNF20‐transfected differentiated adipocytes (Figure 3C). This experiment was performed independently three times, and the quantitative data showed that the levels of RNF20, H2Bub, PPARγ, and CEBPα were decreased significantly (Figure 3D). Taken together, our results revealed that RNF20 knockdown impaired porcine adipocyte differentiation.

To explore the molecular signatures involved in the RNF20‐mediated compromise of adipogenesis in pigs, genome‐wide RNA‐seq was used to analyze the transcriptional profiles between siRNF20‐ and siNC‐transfected mature adipocytes (after 8 days of differentiation). Principal component analysis (PCA) showed that the two groups could be separated clearly, suggesting not only the good quality of the data but also the different molecular networks in the two groups of cells (Figure S2). Our data revealed 744 DEGs with the criteria of a *p*‐value <0.05 and FC ˃ 2.0, and a volcano plot was built based on fold changes and *p*‐values for global visualization of changes in gene expression (Figure 3E). A total of 503 genes were significantly downregulated, while 241 genes were significantly upregulated in siRNF20‐transfected cells. The DEGs were subjected to KEGG enrichment analysis (Table S3). The results showed that the well‐known pathways that regulate adipogenesis, including the PPAR and Wnt signaling pathways, were enriched in downregulated genes. The expression of genes in both pathways was profiled based on the RNA‐seq and qPCR data (Figure S3). In addition, genes involved in focal adhesion, cell adhesion molecules (CAMs) and extracellular matrix (ECM) receptor interactions were found to be significantly downregulated (Figure 3F). Heatmaps were built with integrin subunit genes *(ITGA1, ITGA4, ITGA5, ITGA7, ITGA8*, and *ITGB1*) and CAM‐related genes (*THBS1, THBS2, THBS3, NCAM1, NFASC, NTNG2, NECTIN*, and *VCAN*) based on RNA‐seq data (Figure 3G). The downregulation of these genes in siRNF20‐transfected cells was further validated by qPCR (Figure 3H). As shown in Figure 3I, these CAMs and ECM related genes were closely linked via analysis with the Metascape online tool (https://metascape.org/). Taken together, these data indicate that RNF20 is required for adipogenesis.

### 3.4 Knockdown of the *RNF20* gene inhibited mitotic clonal expansion

**FIGURE 4:**
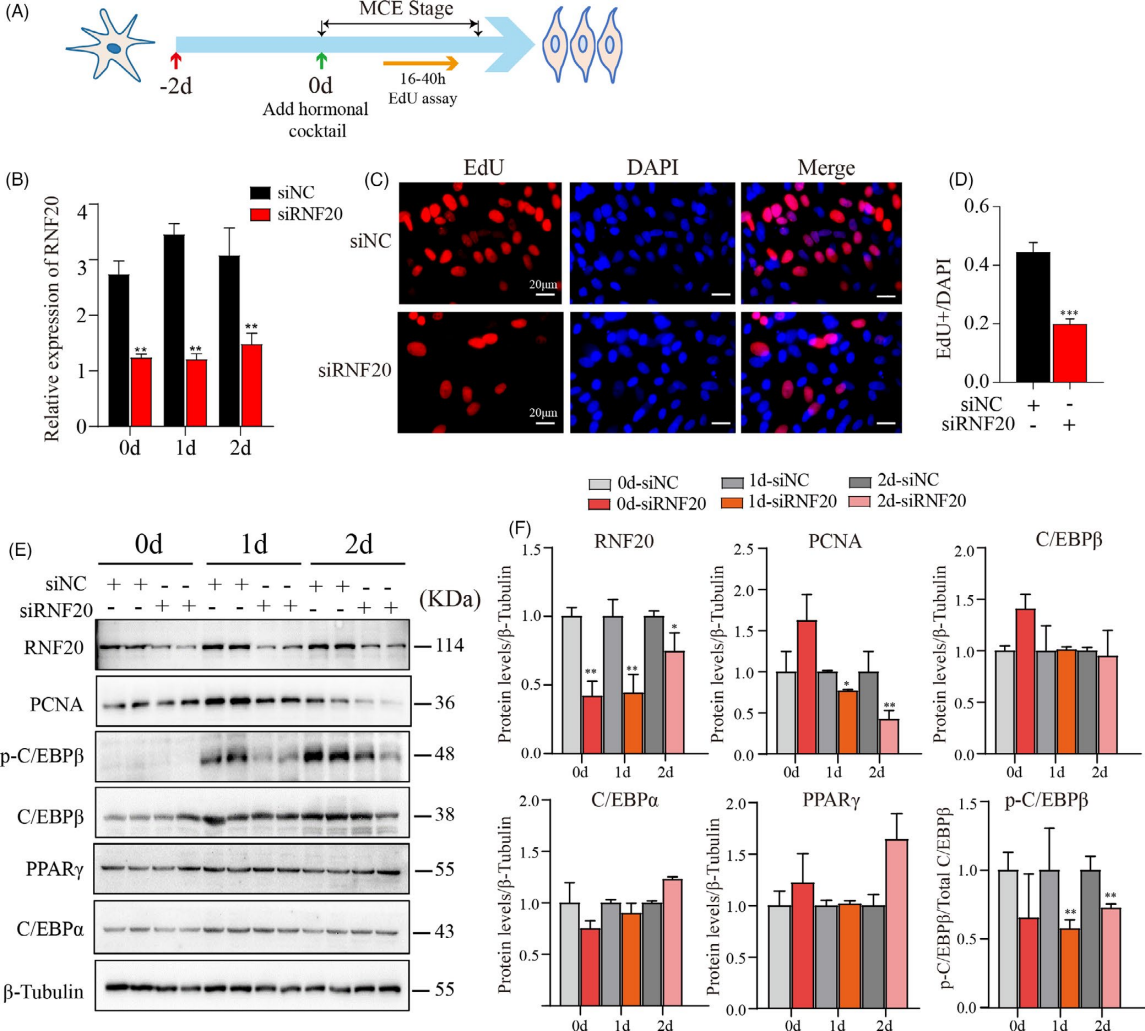
Knockdown of the *RNF20* gene inhibited mitotic clonal expansion of adipogenesis. (A) Schematic diagram for exploring the effect of RNF20 on the MCE phase in this study. The green arrow indicates that preadipocytes were induced with a hormonal cocktail at day 0. Red arrows indicate that cells were transfected with siNC or siRNF20 at day −2. (B) The mRNA expression level of *RNF20* in siNC‐ and siRNF20‐transfected cells was detected by qPCR. Note: The knockdown of *RNF20* was successful in porcine preadipocytes. *18S* was used as an internal control. (C) EdU assay was used to analyze cell proliferation in siNC‐ and siRNF20‐transfected cells. EdU was added at a final concentration of 5 μM after 16 h of differentiation. After a total of 40 h of differentiation, cells were fixed in 4% PFA for 30 min at room temperature and subsequently incubated with reaction cocktail and DAPI. Representative images were selected from at least three independent experiments. (D) Quantitative data from (C). *n* = 16. Results are presented as the mean ± SEM. ***p < 0.01. (E) The expression levels of proteins, including RNF20, proliferation marker PCNA, early adipogenesis markers (C/EBPβ and p‐C/EBPβ) and adipogenic transcription factors (PPARγ and C/EBPα), in siNC‐ and siRNF20‐transfected preadipocytes after day 0, 1 and 2 days of induction. (F) Data quantification of panel (E). Western blots shown are representative of three independent experiments. β‐Tubulin was used as a loading control. Data are presented as the mean ±SEM. *p < 0.05, **p < 0.01

As described above, RNF20 knockdown suppressed the expression levels of genes involved in the focal adhesion, ECM and CAMs signaling pathways, which have been demonstrated to play important roles in the MCE phase.^27^, ^28^, ^29^ To explore the effect of *RNF20* gene on the MCE phase of porcine adipogenesis, siNC or siRNF20 was used to transfect porcine preadipocytes at −2 days, and cells were induced at 0 day and harvested at 0 day, 1 day, and 2 days of induction (Figure 4A). As expected, significantly reduced RNF20 expression at both the mRNA (Figure 4B) and protein levels (Figure 4E,F) was observed in siRNF20‐transfected cells at each time point, while no obvious changes in the expression of PPARγ and C/EBPα were observed in siRNF20‐transfected cells (Figure 4E,F). Porcine preadipocyte proliferation was assessed by an EdU incorporation experiment (Figure 4A). Representative images of EdU staining showed that knockdown of RNF20 led to a dramatic reduction in cell proliferation 40 h after induction of adipogenesis (Figure 4C), and quantitative analysis revealed that the reduction was greater than 50% (Figure 4D). Consistently, the expression level of the cell proliferation marker, proliferating cell nuclear antigen (PCNA), was downregulated at 1 day and 2 days in siRNF20 cells (Figure 4E). It has been reported that the phosphorylation and translocation of C/EBPβ play key roles in activating adipogenic transcription factors during the MCE phase of adipogenesis^30^; therefore, we also measured the level of p‐C/EBPβ in both cells. The levels of p‐C/EBPβ were observed to be compromised in the siRNF20 group (Figure 4E,F).

### 3.5 Knockdown of the *RNF20* gene inhibited the Ras‐Raf‐MEK1/2‐ERK1/2 signaling pathway

**FIGURE 5:**
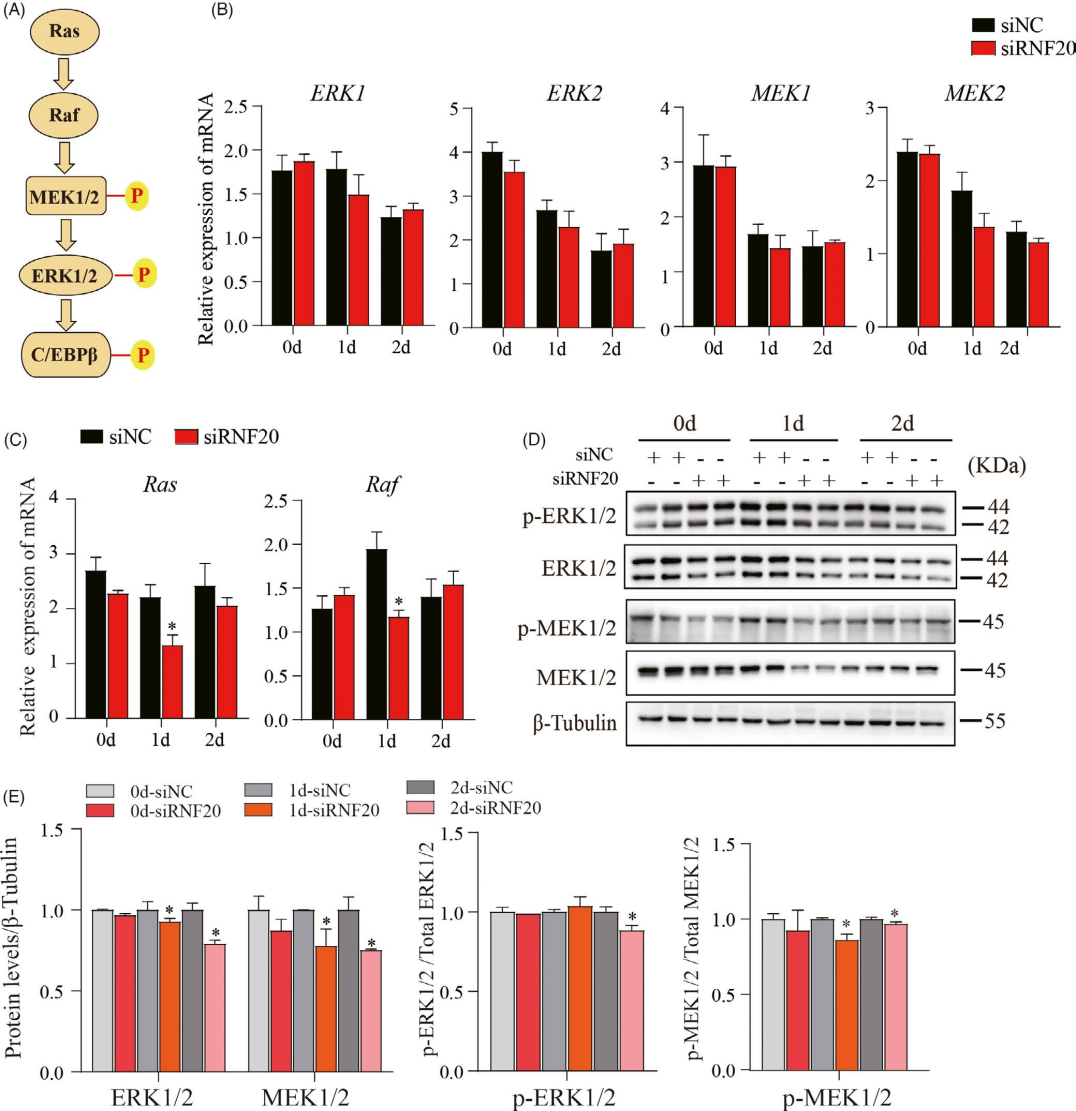
The Ras‐Raf‐MEK1/2‐ERK1/2 signaling pathway was inhibited in RNF20 knockdown cells. (A) The Ras‐Raf‐MEK1/2‐ERK1/2 signaling cascade activated the phosphorylation of C/EBPβ. (B–C) Expression levels of the genes involved in the Ras‐Raf‐MEK1/2‐ERK1/2 signaling pathway. *18S* was used as an internal control. Data are presented as the mean ± SEM, *n* = 3, *p < 0.05. (D) Representative Western blot showed the total protein and phosphorylation levels of ERK1/2 and MEK1/2 in siNC‐ and siRNF20‐transfected cells at day 0, 1 and 2 days after induction. β‐Tubulin was used as a loading control. (E) Data quantification of panel (D). Data are presented as the mean ± SEM. *p < 0.05, **p < 0.01

It has been reported that C/EBPβ is phosphorylated on Thr‐188 by mitogen‐activated protein kinase (MAPK), also known as extracellular‐signal‐regulated kinase (ERK), 2–12 h after adipogenic induction.^31^ In addition, several key signaling components, Ras‐Raf‐MEK1/2‐ERK1/2, have been reported to be involved in the activation cascade of ERKs,^32^, ^33^, ^34^ as shown in Figure 5A. To explore whether the Ras‐Raf‐MEK1/2‐ERK1/2 signaling pathway was affected in siRNF20 knockdown cells during the MCE phase, the expression levels of the *ERK1, ERK2, MEK1, MEK2, Ras*, and *Raf* genes were detected by qPCR at day 0, 1, and 2 days after differentiation. Our results showed that only the *Ras* and *Raf1* genes were reduced in siRNF20‐transfected cells at 1 day after induction (Figure 5B,C). However, we observed that the phosphorylation and total protein levels of ERK1/2 and MEK1/2 were significantly decreased in siRNF20‐transfected cells at either 1 day and/or 2 days after differentiation (Figure 5D,E). Taken together, these data suggest that knockdown of RNF20 suppressed p‐C/EBPβ via the Ras‐Raf‐MEK1/2‐ERK1/2 signaling pathway.

## 4 DISCUSSION

Genome editing tools have provided efficient ways to produce genetically modified pigs for the dramatic and rapid improvement of economic traits. However, few agriculturally useful genes have been identified and discovering porcine genes that regulate the important agricultural traits is prerequisite for molecular design breeding in pigs. In this study, we explored the effect of RNF20 on adipogenesis in pigs, as a livestock possesses strong fat deposition ability. A significant higher expression level of RNF20 was observed in backfat tissues from fat‐type pigs, which was consistent with the observation that a higher RNF20 protein level was found in fat tissues from high‐fat diet‐fed mice than in those from chow diet‐fed mice.^23^ Both studies suggested a positive correlation of the RNF20 expression level with fat deposition. This positive effect has been further confirmed in *Rnf20*‐defective (both *Rnf20* ^+/−^,^23^ and adipocyte‐specific *Rnf20* knockout^24^) mouse models, in which fat mass was significantly reduced with smaller adipocytes under both chow diet and high‐fat diet conditions compared with wild‐type littermates.^23^, ^24^ At this point, it is important to investigate the expression levels of RNF20 in fat tissues from more fat‐type and lean‐type pig populations before it can be used as a useful marker for fat deposition. Together with the observation of high expression level of RNF20 in differentiated adipocytes, suggested that this protein plays the key role both in late differentiation stage of adipogenesis and in maintenance of mature adipocytes.

It has been reported that RNF20 regulates hepatic lipid metabolism via ubiquitination and degradation of the key transcription factor SREBP1c^22^ and acts as a transcriptional coactivator for PPARγ by promoting NCoR1 degradation in adipocytes.^23^ Unlike polyubiquitination, which mainly serves to send proteins for degradation by the proteasome, RNF20 has been implicated as responsible for monoubiquitination of histone H2B lysine 120, which is regarded as a prerequisite for trimethylation of histone 3 lysine 4 (H3K4me3) and histone 3 lysine 79 (H3K79me3)^35^ and regulates gene functions by recruiting various H2Bub‐specific readers or preventing the binding of others in many cellular processes.^36^ Here, although we observed the reduced expression levels of H2Bub (Figure 3C), H3K4me3, and H3K79me3 (Figure S4A,B) in siRNA20‐transfected cells, whether RNF20 affects porcine adipogenesis via ubiquitination and degradation of SREBP1c or NCoR1, or via the RNF20‐H2Bub‐H3K4me3 axis needs to be further investigated.

Consistent with the observations in 3T3‐L1 cells,^23^, ^24^ porcine preadipocyte differentiation efficiency was compromised via knockdown of RNF20. Adipogenesis occurs in several stages, and as the key event of the MCE stage, the activity of C/EBPβ was observed to be decreased in RNF20 knockdown cells (Figure 4E). The fact that C/EBPβ functions in the early step of cell fate commitment in adipogenesis suggest the involvement of RNF20 in cell commitment. At this point, we believed that RNF20 is critical to porcine adipogenesis, not only at the terminal differentiation stage but also at the MCE stage.

Although our data suggested that RNF20 affects p‐C/EBPβ by regulating the Ras‐Raf‐MEK1/2‐ERK1/2 signaling pathway, how RNF20 exerts its roles on CAM‐ and ECM‐related genes and the Ras‐Raf‐MEK1/2‐ERK1/2 signaling cascade in the MCE phase and terminal differentiation of adipogenesis remain unknown and need to be further investigated. It has been reported that RNF20 regulates gene expression by modulating chromatin structure,^37^, ^38^ we proposed that the decreased expression of *Ras* and *Raf* in RNF20 knockdown adipocytes might be due to the tighter chromatin structure.

Ras is a member of the superfamily of small GTPase and linked to cell adhesion, cell cycle progression, and cell proliferation pathway.^34^ Cell adhesion plays a role in regulation of adipogenesis because cell rounding is an important first step for proper preadipocytes differentiation.^27^ The downregulation of *Ras*, *Raf*, CAMs, and ECM‐related genes in RNF20 knockdown cells hints that RNF20 may affect the differentiation through regulating cell adhesion process and this hypothesis needs to be further studied.

In summary, the present study provides evidence that RNF20 is an important regulator of porcine adipogenesis, acting to inhibit the activity of a key transcription factor in the MCE phase of adipogenic differentiation, C/EBPβ. Further exploration of the detailed mechanisms of RNF20 in porcine adipogenesis should lead to the identification of additional targets for the selection of fat deposition traits in pigs.

## CONFLICT OF INTEREST

We declare that no conflict of interest exists.

## AUTHOR CONTRIBUTIONS

YW and JZ designed the project; YZ, JP, XL, LL, SY, CT, and CC collected and analyzed the data; YW and YZ wrote the manuscript. All authors reviewed the manuscript.

## Supporting information

Fig S1

Fig S2

Fig S3

Fig S4

Tab S1

Tab S2

Tab S3

## Data Availability

The data that support that the findings of this study are available on request from the corresponding author. The data are not publicly available due to privacy or ethical restrictions.

## References

[1] Poklukar K , Candek‐Potokar M , Batorek Lukac N , Tomazin U , Skrlep M . Lipid deposition and metabolism in local and modern pig breeds: a review. Animals (Basel). 2020;10(3):424. 10.3390/ani10030424 PMC7142902 32138208

[2] Cornelius P , MacDougald OA , Lane MD . Regulation of adipocyte development. Annu Rev Nutr. 1994;14:99‐129. 7946535 10.1146/annurev.nu.14.070194.000531

[3] MacDougald OA , Lane MD . Transcriptional regulation of gene expression during adipocyte differentiation.pdf. Annu Rev Biochem. 1995;64:345‐373. 7574486 10.1146/annurev.bi.64.070195.002021

[4] Tang Q‐Q , Otto TC , Lane MD . Mitotic clonal expansion a synchronous process required for adipogenesis. PNAS. 2002;100(1):44‐49. 12502791 10.1073/pnas.0137044100 PMC140878

[5] Tang QQ , Otto TC , Lane MD . Mitotic clonal expansion: a synchronous process required for adipogenesis. Proc Natl Acad Sci USA. 2003;100(1):44‐49. 12502791 10.1073/pnas.0137044100 PMC140878

[6] Rosen ED , MacDougald OA . Adipocyte differentiation from the inside out. Nat Rev Mol Cell Biol. 2006;7(12):885‐896. 17139329 10.1038/nrm2066

[7] Gregoire FM , Smas CM , Sul HS . Understanding adipocyte differentiation. Physiol Rev. 1998;78(3):783‐809. 9674695 10.1152/physrev.1998.78.3.783

[8] Tang Q‐Q , Lane MD . Activation and centromeric localization of CCAAT enhancer‐binding proteins during the mitotic clonal expansion of adipocyte differentiation. Genes Dev. 1999;13:2231‐2241. 10485846 10.1101/gad.13.17.2231 PMC316997

[9] Hwang WW , Venkatasubrahmanyam S , Ianculescu AG , Tong A , Boone C , Madhani HD . A conserved RING finger protein required for histone H2B monoubiquitination and cell size control. Mol Cell. 2003;11(1):261‐266. 12535538 10.1016/s1097-2765(02)00826-2

[10] Wood A , Krogan NJ , Dover J , et al. Bre1, an E3 ubiquitin ligase required for recruitment and substrate selection of Rad6 at a promoter. Mol Cell. 2003;11(1):267‐274. 12535539 10.1016/s1097-2765(02)00802-x

[11] Trujillo KM , Osley MA . A role for H2B ubiquitylation in DNA replication. Mol Cell. 2012;48(5):734‐746. 23103252 10.1016/j.molcel.2012.09.019 PMC3525772

[12] Nakamura K , Kato A , Kobayashi J , et al. Regulation of homologous recombination by RNF20‐dependent H2B ubiquitination. Mol Cell. 2011;41(5):515‐528. 21362548 10.1016/j.molcel.2011.02.002

[13] Chernikova SB , Dorth JA , Razorenova OV , Game JC , Brown JM . Deficiency in Bre1 impairs homologous recombination repair and cell cycle checkpoint response to radiation damage in mammalian cells. Radiat Res. 2010;174(5):558‐565. 20738173 10.1667/RR2184.1 PMC2988074

[14] Jung I , Kim SK , Kim M , et al. H2B monoubiquitylation is a 5'‐enriched active transcription mark and correlates with exon‐intron structure in human cells. Genome Res. 2012;22(6):1026‐1035. 22421545 10.1101/gr.120634.111 PMC3371706

[15] Wu C , Cui Y , Liu X , Zhang F , Lu LY , Yu X . The RNF20/40 complex regulates p53‐dependent gene transcription and mRNA splicing. J Mol Cell Biol. 2020;12(2):113‐124. 31152661 10.1093/jmcb/mjz045 PMC7109600

[16] Pirngruber J , Shchebet A , Schreiber L , et al. CDK9 directs H2B monoubiquitination and controls replication‐dependent histone mRNA 3'‐end processing. EMBO Rep. 2009;10(8):894‐900. 19575011 10.1038/embor.2009.108 PMC2726677

[17] Vitaliano‐Prunier A , Babour A , Herissant L , et al. H2B ubiquitylation controls the formation of export‐competent mRNP. Mol Cell. 2012;45(1):132‐139. 22244335 10.1016/j.molcel.2011.12.011 PMC3259529

[18] Karpiuk O , Najafova Z , Kramer F , et al. The histone H2B monoubiquitination regulatory pathway is required for differentiation of multipotent stem cells. Mol Cell. 2012;46(5):705‐713. 22681891 10.1016/j.molcel.2012.05.022

[19] Vethantham V , Yang Y , Bowman C , et al. Dynamic loss of H2B ubiquitylation without corresponding changes in H3K4 trimethylation during myogenic differentiation. Mol Cell Biol. 2012;32(6):1044‐1055. 22252316 10.1128/MCB.06026-11 PMC3295016

[20] Liang Q , Xia W , Li W , Jiao J . RNF20 controls astrocytic differentiation through epigenetic regulation of STAT3 in the developing brain. Cell Death Differ. 2018;25(2):294‐306. 28984873 10.1038/cdd.2017.157 PMC5762844

[21] Ren P , Sheng Z , Wang Y , et al. RNF20 promotes the polyubiquitination and proteasome‐dependent degradation of AP‐2alpha protein. Acta Biochim Biophys Sin (Shanghai). 2014;46(2):136‐140. 24374663 10.1093/abbs/gmt136

[22] Lee JH , Lee GY , Jang H , Choe SS , Koo SH , Kim JB . Ring finger protein20 regulates hepatic lipid metabolism through protein kinase A‐dependent sterol regulatory element binding protein1c degradation. Hepatology. 2014;60(3):844‐857. 24425205 10.1002/hep.27011 PMC4258077

[23] Jeon YG , Lee JH , Ji Y , et al. RNF20 functions as a transcriptional coactivator for PPARgamma by promoting NCoR1 degradation in adipocytes. Diabetes. 2020;69(1):20‐34. 31604693 10.2337/db19-0508

[24] Liang X , Tao C , Pan J , et al. Rnf20 deficiency in adipocyte impairs adipose tissue development and thermogenesis. Protein Cell. 2021;12(6):475‐492. 32797353 10.1007/s13238-020-00770-2 PMC8160045

[25] Zhao Y , Yang S , Wang Y , Tao C . Molecular characterization, expression profiling, and SNP analysis of the porcine RNF20 gene. Animals (Basel). 2020;10(5):888. 10.3390/ani10050888 PMC7278386 32443664

[26] Lin J , Cao C , Tao C , et al. Cold adaptation in pigs depends on UCP3 in beige adipocytes. J Mol Cell Biol. 2017;9(5):364‐375. 28486585 10.1093/jmcb/mjx018

[27] Szabo E , Feng T , Dziak E , Opas M . Cell adhesion and spreading affect adipogenesis from embryonic stem cells: the role of calreticulin. Stem Cells. 2009;27(9):2092‐2102. 19544411 10.1002/stem.137

[28] Xu B , Ju Y , Song G . Role of p38, ERK1/2, focal adhesion kinase, RhoA/ROCK and cytoskeleton in the adipogenesis of human mesenchymal stem cells. J Biosci Bioeng. 2014;117(5):624‐631. 24331979 10.1016/j.jbiosc.2013.10.018

[29] Croissandeau G , Chretien M , Mbikay M . Involvement of matrix metalloproteinases in the adipose conversion of 3T3‐L1 preadipocytes. Biochem J. 2002;364:739‐746. 12049638 10.1042/BJ20011158 PMC1222623

[30] Tang Q‐Q , Otto TC , Lane MD . CCAAT enhancer‐binding protein beta is required for mitotic clonal expansion during adipogenesis. PNAS. 2002;100(3):850‐855. 10.1073/pnas.0337434100 PMC298690 12525691

[31] Guo L , Li X , Tang QQ . Transcriptional regulation of adipocyte differentiation: a central role for CCAAT/enhancer‐binding protein (C/EBP) beta. J Biol Chem. 2015;290(2):755‐761. 25451943 10.1074/jbc.R114.619957 PMC4294498

[32] Davis S , Laroche S . Mitogen‐activated protein kinase/extracellular regulated kinase signalling and memory stabilization: a review. Genes Brain Behav. 2006;5(Suppl 2):61‐72. 16681801 10.1111/j.1601-183X.2006.00230.x

[33] Lewis TS , Shapiro PS , Ahn NG . Signal transduction through MAP kinase cascades. Adv Cancer Res. 1998;74:49‐139. 9561267 10.1016/s0065-230x(08)60765-4

[34] Santarpia L , Lippman SM , El‐Naggar AK . Targeting the MAPK‐RAS‐RAF signaling pathway in cancer therapy. Expert Opin Ther Targets. 2012;16(1):103‐119. 22239440 10.1517/14728222.2011.645805 PMC3457779

[35] Soares LM , Buratowski S . Histone crosstalk: H2Bub and H3K4 methylation. Mol Cell. 2013;49(6):1019‐1020. 23541037 10.1016/j.molcel.2013.03.012 PMC3753804

[36] Fuchs G , Oren M . Writing and reading H2B monoubiquitylation. Biochim Biophys Acta. 2014;1839(8):694‐701. 24412854 10.1016/j.bbagrm.2014.01.002

[37] Xu Z , Song Z , Li G , et al. H2B ubiquitination regulates meiotic recombination by promoting chromatin relaxation. Nucleic Acids Res. 2016;44(20):9681‐9697. 27431324 10.1093/nar/gkw652 PMC5175339

[38] Fierz B , Chatterjee C , McGinty RK , Bar‐Dagan M , Raleigh DP , Muir TW . Histone H2B ubiquitylation disrupts local and higher‐order chromatin compaction. Nat Chem Biol. 2011;7(2):113‐119. 21196936 10.1038/nchembio.501 PMC3078768

