# Supplementary figures and images for: RNF20 affects porcine adipocyte differentiation via regulation of mitotic clonal expansion
Source: Cell Prolif. 2021 Oct 14;54(12):e13131. doi: 10.1111/cpr.13131 (PMC8666272; doi:10.1111/cpr.13131)

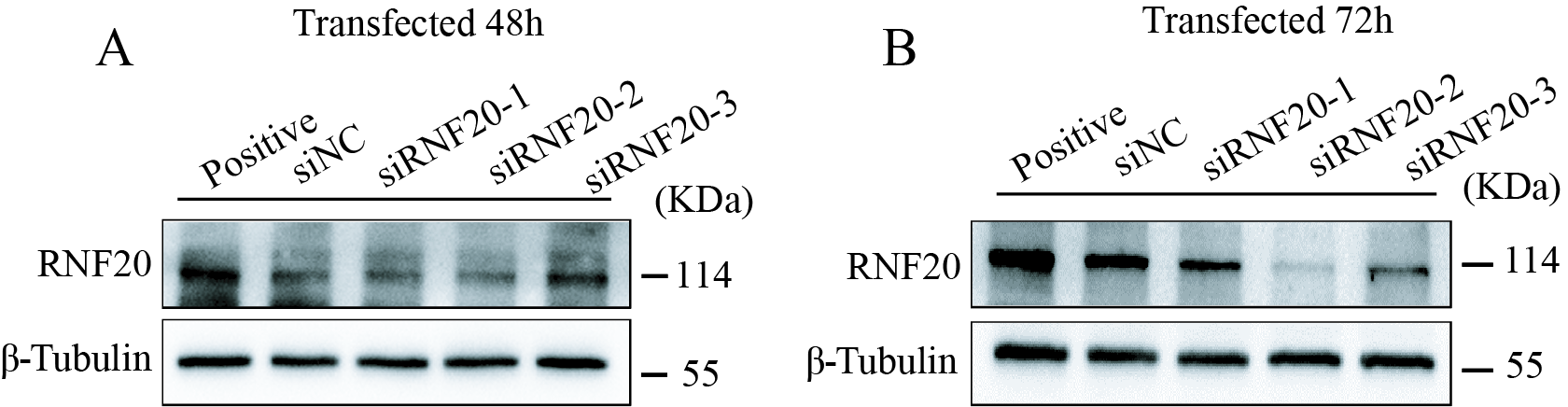

Supplement: Supplementary file 1 — Fig S1 [file CPR-54-e13131-s004.tif]

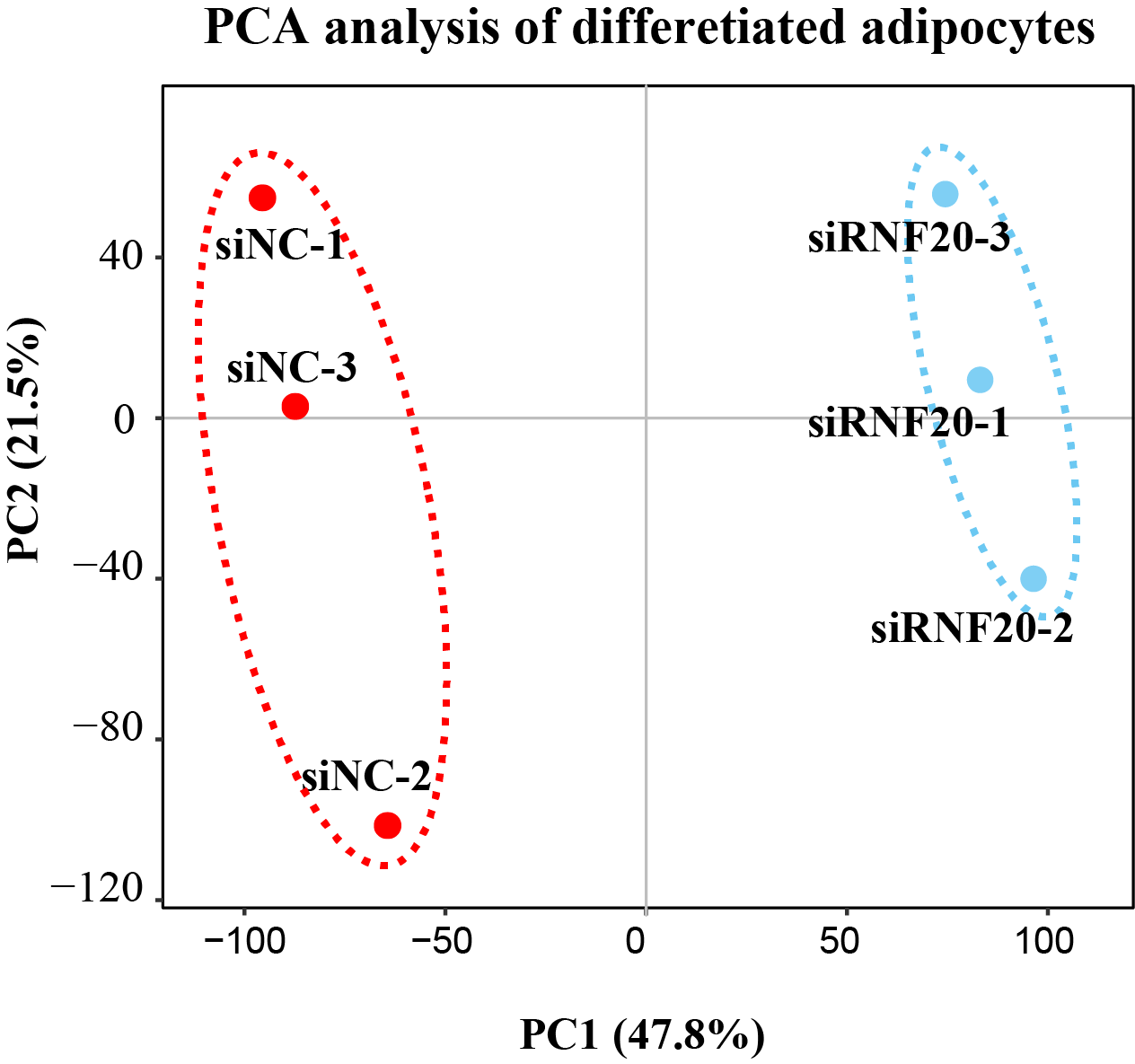

Supplement: Supplementary file 2 — Fig S2 [file CPR-54-e13131-s007.tif]

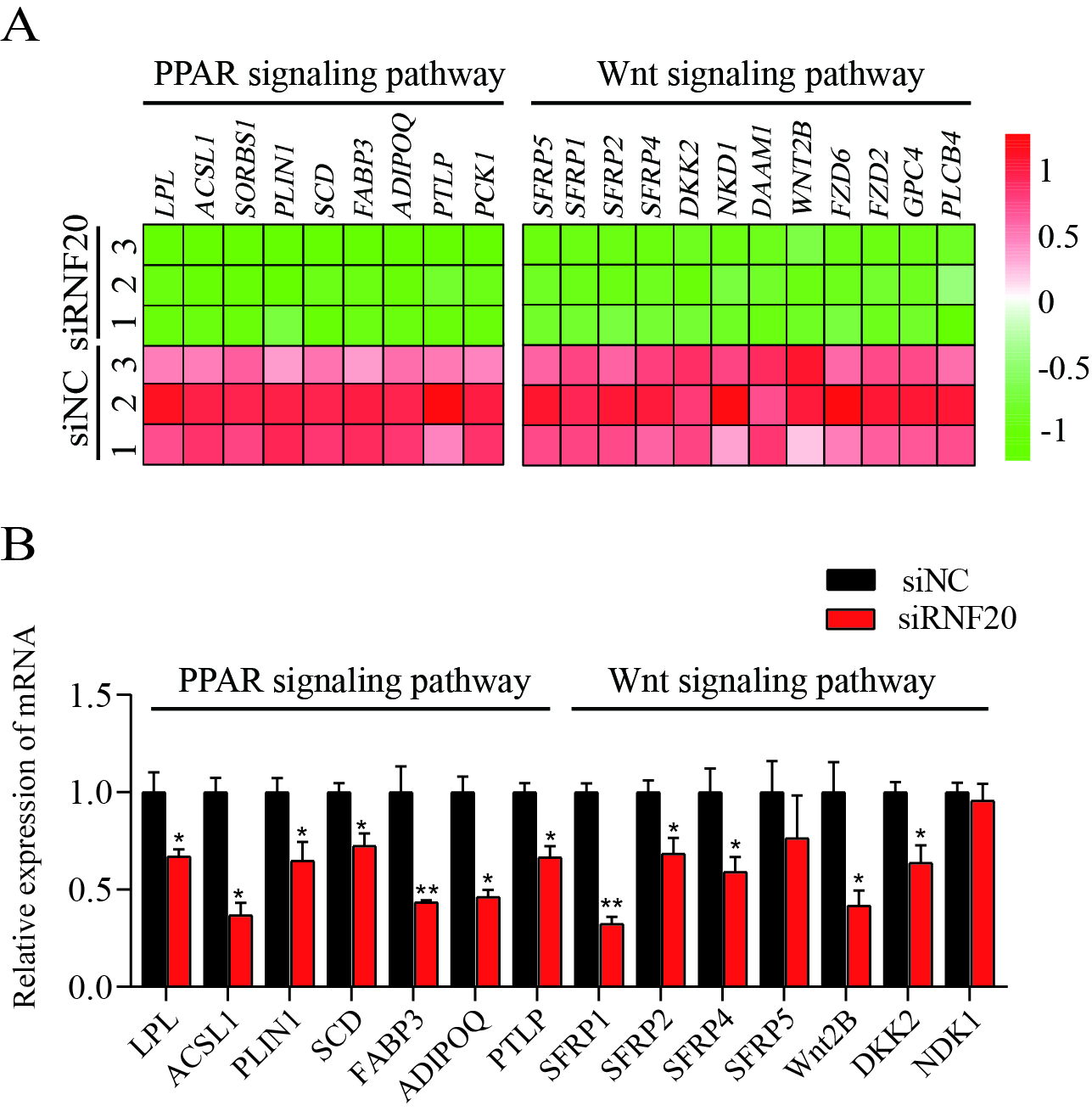

Supplement: Supplementary file 3 — Fig S3 [file CPR-54-e13131-s003.tif]

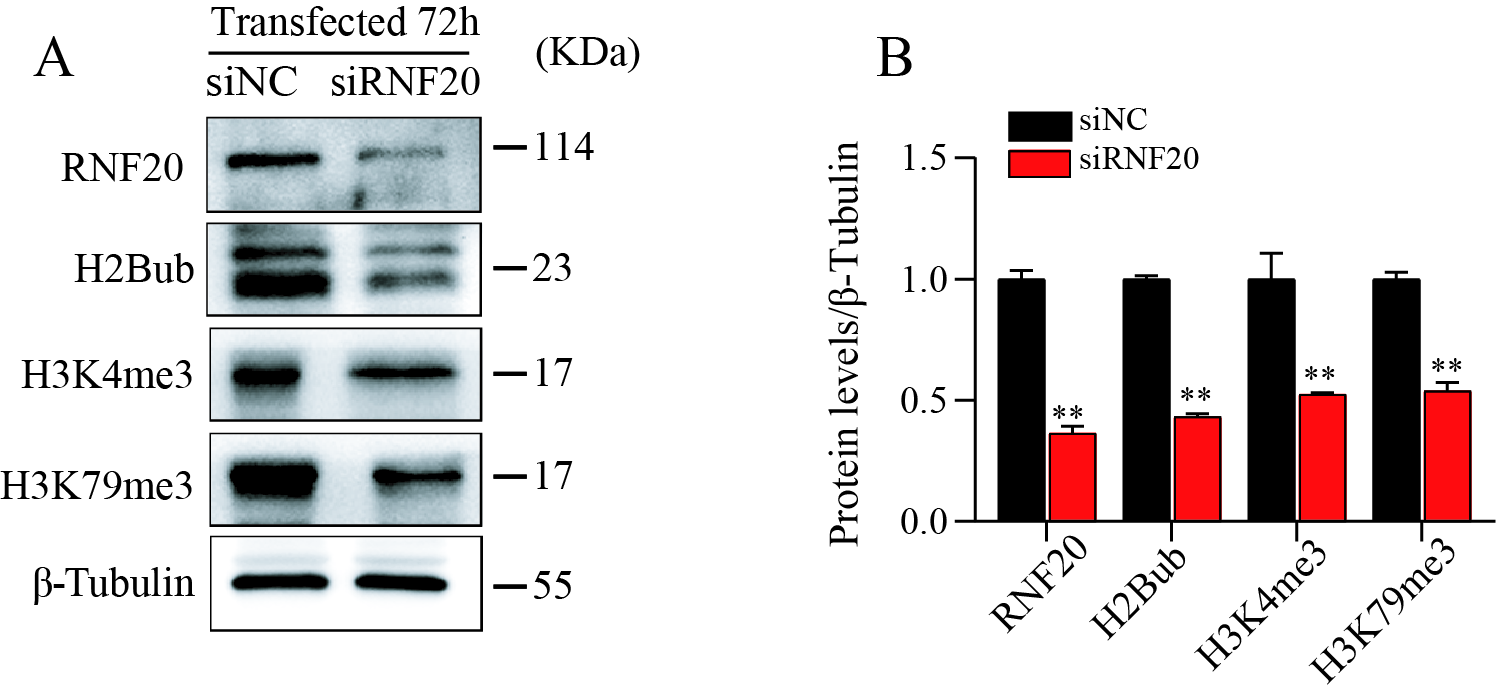

Supplement: Supplementary file 4 — Fig S4 [file CPR-54-e13131-s005.tif]
